# Low‐molecular weight heparin prevents portal vein system thrombosis after splenectomy: a systematic review and meta‐analysis

**DOI:** 10.1111/ans.15865

**Published:** 2020-04-27

**Authors:** Ming Yang, Jinlong Liu

**Affiliations:** ^1^ Department of Hepatobiliary Surgery Affiliated Hospital of ChengDe Medical University Chengde China

**Keywords:** low‐molecular weight heparin, portal vein system thrombosis, splenectomy

## Abstract

**Background:**

The aim of this study was to evaluate the safety and efficacy of low‐molecular weight heparin (LMWH) in the prevention of portal vein system thrombosis (PVST) after splenectomy.

**Methods:**

A systematic search was performed using PubMed, EMBASE, Springer and Cochrane Library databases to screen out studies comparing the prognoses between post‐splenectomy patients treated with and without LMWH. The incidences of PVST and bleeding complications were used as parameters to assess the effect of LMWH.

**Results:**

Six articles met the selection criteria and were included in this study. A total of 740 patients were involved in these six articles, including 336 patients treated with LMWH (LMWH group) and 385 patients not treated with LMWH (control group). The incidence of PVST in the LMWH group was significantly lower than that in the control group (relative risk 1.782 (1.449–2.192); *P* = 0.285; *I* ^2^ = 19.7%), while the incidence of post‐operative bleeding in the LMWH group was significantly higher (relative risk 0.592 (0.195–1.799); *P* = 0.817; *I* ^2^ = 0.0%).

**Conclusion:**

LMWH might decrease the incidence of PVST after splenectomy without a potential risk of bleeding.

## Graphical abstract

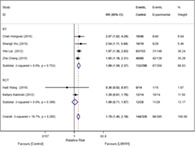

The aim of this study was to evaluate the safety and efficacy of low‐molecular weight heparin in the prevention of portal vein system thrombosis after splenectomy.

## Introduction

Splenectomy is a common operation used in the treatment of splenic trauma, inflammatory splenic diseases and hypersplenism. In particular, splenectomy is most frequently used to treat diseases associated with hypersplenism, such as portal hypertension (PH) with hypersplenism and immune thrombocytopenic purpura. Given the broad application of splenectomy in China owing to its large population of hepatitis patients, great attention has been paid to the complications of splenectomy, with portal vein system thrombosis (PVST) being the most severe one.

PVST refers to a thrombus formed in the trunk of portal vein or portal branch including superior mesenteric vein, splenic vein (SV) and inferior mesenteric vein.^1^ One prospective study has shown the high incidence of symptomatic PVST in 19% of cases after laparoscopic splenectomy.^2^ As a potentially fatal complication, PVST can cause the thrombus to spread to the superior mesenteric vein and lead to intestinal infarction, subsequently inducing peritoneum inflammation, shock or even death.^3^, ^4^ Although several studies^5^, ^6^ have demonstrated the mechanisms underlying PVST formation and related risk factors, there is still no effective way to prevent PVST. Nevertheless, post‐operative anticoagulation has been applied as a preventive measure. Various anticoagulation drugs including warfarin, aspirin, antithrombin III and low‐molecular weight heparin (LMWH) have been used in patients after splenectomy, with LMWH being the most frequently adopted one in recent years. At the same time, the preventive role and the safety of LMWH have been under dispute.^7^, ^8^ Studies have shown that the risk of bleeding should be considered prior to the use of LMWH immediately after splenectomy,^9^ especially in cirrhotic patients with coagulation disorders.

Therefore, the aim of this meta‐analysis was to clarify the value of prophylactic LMWH treatment in reducing PVST after splenectomy and assess the risk of LMWH‐induced bleeding.

## Methods

### Literature search

A systematic review was performed independently by two authors of this study (Dr JL and Dr MY) using PubMed, EMBASE, Springer and Cochrane Library databases, so as to screen out comparative studies evaluating the efficacy of different anticoagulation strategies (with and without LMWH) in preventing PVST in patients undergoing splenectomy. The literature search was restricted to comparative studies (prospective or retrospective) written in English and published as of 31 July 2018. This study protocol followed the Preferred Reporting Items for Systematic Reviews and Meta‐Analyses statement.^10^

The search was based on the combinations of the following keywords: ((((splenectomy) and heparin) and thrombus)) and anticoagulation. The reference list of screened articles was also checked for potential hits.

### Study selection

The two reviewers mentioned above also independently performed eligibility assessment and screening. All titles and abstracts were screened for relevance of each study to this review. The inclusion criteria of the screening were: (i) randomized or comparative studies, regardless of the number of patients in each arm, that compared the effects of different anticoagulation strategies (with and without LMWH) in preventing PVST in patients undergoing splenectomy; (ii) the dose and timing of post‐splenectomy anticoagulation treatment were clearly shown; (iii) outcome information, including the incidence of PVST, was available; and (iv) randomized controlled trials (RCTs) with an evaluation of three or more ‘yes’ and retrospective non‐randomized trials (RTs) with a cumulative quality of literature score ≥7.

The exclusion criteria of the screening were: (i) without a control group; (ii) incomplete raw data for the purpose of this research; (iii) only involving animals or cells; (iv) reviews, study protocols, comments or case reports; and (v) studies unrelated to the prevention of post‐splenectomy PVST.

If the two reviewers disagreed about the inclusion/exclusion decision for a given study, a consensus meeting was held to decide its eligibility.

### Data extraction

The two reviewers examined relevant texts, tables and figures to extract data from articles that have been included in this study. The following information was collected: (i) name of the first author, year of publication, type of study, operation method, bleeding location and detection medium; (ii) demographics (sample size in each group, age, gender, Child‐Pugh classification, aetiology, preoperative platelet count, portal vein diameter and SV diameter); (iii) anticoagulants used in the study; and (iv) primary outcome, that, post‐operative incidence of PVST, and secondary outcome, that is, the incidence of post‐operative bleeding. The data forms from the two authors were compared, and were resolved via consensus between the two authors.

### Quality assessment of evidence

The methodological quality of RCTs was assessed using the method described in the Cochrane Handbook for Systematic Reviews of Interventions.^11^ Risk of bias for each eligible RCTs was determined by six items: (i) the method of random allocation; (ii) the concealment of allocation; (iii) the blinding method; (iv) the integrity of outcome data; (v) the outcome data of selective reports; and (vi) other bias sources. The evaluation results for each item include: ‘yes’, ‘no’ and ‘unclear’. The studies with three or more yes were considered high quality and less than three yes were recognized as low quality. The methodological quality of retrospective non‐RTs was assessed using the modified Newcastle–Ottawa scale.^12^ This tool was conducted to evaluate the RTs across three components: (i) patient selection (0–4 points); (ii) comparability (0–2 points); and (iii) outcome (0–3 points). The RTs with ≥7 points could be recognized as high quality. Differences in evaluation regarding bias of studies were resolved through discussion and consensus.

### Statistical analysis

Statistical analysis was performed using State21.0 (StataCorp, College Station, TX, USA). The incidences of post‐operative PVST and post‐operative bleeding were treated as dichotomous data and were analysed using pooled relative risk (RR). Statistical significance was set at *P* < 0.05. Heterogeneity was assessed using *I* ^2^ and chi‐squared test, and was deemed significant if *I* ^2^ >50% and/or *P* < 0.05. A random‐effects model and a fixed‐effects model were used for inter‐study heterogeneity (*I* ^2^ > 50%) or homogeneity,^13^ respectively. Finally, Egger's test was used to evaluate publication bias of the studies included.

## Results

### Literature search results

The Preferred Reporting Items for Systematic Reviews and Meta‐Analyses flow diagram of the included trials is shown in Figure S1. The literature search yielded 442 potentially relevant articles, with 439 identified through database searching, with three additional records identified through other sources. One hundred and thirty‐two were excluded from analysis after the first screening based on abstracts and titles, leaving 265 available for further full‐text review. After carefully reading the full‐text articles, 259 were excluded for reasons shown in Figure S1. Finally, six unique studies,^14^, ^15^, ^16^, ^17^, ^18^, ^19^ with suitable data were included and analysed in the meta‐analysis.

### Characteristics of the included studies and quality assessment

Four retrospective non‐RTs^14^, ^16^, ^18^, ^19^ and two RCTs,^14^, ^15^ which involved 385 patients treated with LMWH (LMWH group) and 326 patients not treated with LMWH (control group), were picked out for further analysis. The characteristics of each study are displayed in Table S1. The summary of their clinical data is displayed in Table S2. These six studies were published in 2012–2015 in Canada (*n* = 1), China (*n* = 4) and Japan (*n* = 1). In the five studies published in China and Japan, the patients were diagnosed with liver cirrhosis, PH and/or hypersplenism.^15^, ^16^, ^17^, ^18^, ^19^ In the study published in Canada, the patients were diagnosed with haematological diseases.^14^

The results of the quality assessment are summarized as follows: two RCTs^14^, ^15^ obtained four yes. Of four RTs, two studies^16^, ^18^ received a score of 8 and two studies^17^, ^19^ received a score of 7. The overall quality of included studies was good.

### Primary outcome: incidence of PVST

Colour Doppler ultrasound was performed in these six studies to monitor the formation of PVST in the main trunk or branches of the portal vein system.^14^, ^15^, ^16^, ^17^, ^18^, ^19^ In the six studies,^14^, ^15^, ^16^, ^17^, ^18^, ^19^ 711 patients (LMWH *n* = 385, control *n* = 326) reported post‐operative PVST. The incidence of thrombosis in the LMWH group was 25.45% (*n* = 98) and was significantly lower than that in the control group (44.17% (*n* = 144)) (RR 1.782 (1.449–2.192); *P* = 0.285; *I* ^2^ = 19.7%; Fig. S2).

Subgroup analysis was performed according to the study design of these articles (RCTs or retrospective non‐randomized studies). The two RCTs^14^, ^15^ demonstrated no difference between LMWH and control groups in the incidence of PVST (RR 1.093 (0.714–1.673); *P* = 0.399; *I* ^2^ = 0%; Fig. S2). However, the subgroup analysis of the four non‐randomized studies^16^, ^17^, ^18^, ^19^ showed a significant reduction in the incidence of PVST in the LMWH group compared with that in the control group (RR 1.887 (1.505–2.366); *P* = 0.753; *I* ^2^ = 0.0%; Fig. S2).

### Secondary outcome: incidence of post‐operative bleeding

Post‐operative bleeding was assessed in the five studies^14^, ^16^, ^17^, ^18^, ^19^ (683 patients) shown in Figure S3. The incidence of total bleeding events in the LMWH group was 2.4% (9/371). The incidence of total bleeding events in the control group was 1.3% (4/312). The LMWH group showed gastrointestinal bleeding (*n* = 2), abdominal bleeding (*n* = 3), epistaxis (*n* = 1), subcutaneous ecchymosis (*n* = 1) and bleeding at the surgical site (*n* = 2). In the pooled analysis, the incidence of bleeding was not significantly higher in the LMWH group (RR 0.592 (0.195–1.799); *P* = 0.817; *I* ^2^ = 0.0%; Fig. S3).

### Risk of bias across studies

To evaluate publication bias, we performed Egger's test. The Egger's test gave a *P*‐value of 0.757 for the incidence of PVST and a *P*‐value of 0.232 for the incidence of post‐operative bleeding, indicating no evidence of publication bias (Figs [Link], [Link]).

## Discussion

Splenectomy is mainly used for the treatment of liver cirrhosis in patients with PH and thrombocytopenia caused by autoimmune diseases such as immune thrombocytopenic purpura.^20^, ^21^ However, the reported incidence of post‐splenectomy PVST ranges from 5%^22^ to 52%.^8^ As an alarming and potentially life‐threatening complication of splenectomy, PVST can cause severe intestinal ischaemic necrosis and variceal bleeding.^23^ The complex physiological mechanisms underlying PVST may be related to the following factors: (i) a surged platelet count; (ii) unstable haemodynamics of the portal system; (iii) a cirrhotic and hypercoagulable state of the body; and (iv) intraoperative injury to the SV.^24^, ^25^ In addition, the diameters of splenic and portal veins are considered as major predisposing factors of post‐splenectomy PVST.^26^ For example, after the spleen is removed, the rate of blood flow in the SV is suddenly reduced to induce thrombus formation.^27^ As these pathophysiological mechanisms are closely related to the coagulation and anticoagulation system,^8^ various antithrombotic drugs such as warfarin, aspirin, antithrombin III and LMWH have been used to prevent post‐splenectomy PVST. Clinicians have found that prophylactic anticoagulation and anti‐agglutination therapies can effectively prevent post‐operative PVST in liver cirrhosis patients undergoing splenectomy.^28^ One RCT proposed a dalteparin‐based antithrombotic regimen to prevent venous thrombosis after most abdominal surgeries,^29^ while several previous studies^30^, ^31^ regarded LMWH as a helpful treatment to prevent post‐splenectomy PVST. Nevertheless, it still remains controversial as which medicine is the most beneficial to post‐splenectomy patients.^32^

In Cheng's^33^ meta‐analysis, antithrombin III was reported as more suitable than LMWH for cirrhotic patients. In Xingshun's^27^ meta‐analysis, a pharmacological prophylaxis treatment was recommended for decreasing the incidence of post‐splenectomy PVST. However, the authors failed to describe the details of medicines used in the studies. Ning's^34^ meta‐analysis asserted that early prophylactic anticoagulation treatment could reduce the incidence of PVST. Nevertheless, five out of seven included studies used LMWH, and only two used ATIII. Theoretically, there are some differences between antithrombin III and LMWH. LMWH can inhibit the activation of thrombin and formation of thrombosis by suppressing the function of factor Xa.^35^ Jianying's^36^ meta‐analysis also lacked the detailed information of the anticoagulant used in the study. In Wei's meta‐analysis,^37^ LMWH was effective to prevent post‐splenectomy PVST. However, their research and our research had some differences. To find highly relevant literature, we limited the language to English and the search deadline to July 2018. Finally, we included six studies discussing the effect and safety of LMWH in the prevention of post‐splenectomy PVST.

According to the analysis of the six included articles,^14^, ^15^, ^16^, ^17^, ^18^, ^19^ the incidence of post‐splenectomy PVST was decreased in the LMWH group. In addition, in the RCT subgroup, we found that the rate of post‐splenectomy thrombosis showed no significant difference in patients treated with heparin (RR 1.782 (1.449–2.192); *P* = 0.285; *I* ^2^ = 19.7%). For the group of retrospective non‐randomized studies, we found that the LMWH group had a lower incidence of thrombosis (31% versus 54%). Nevertheless, more RCT studies are needed to verify the result of this study. In addition, our meta‐analysis showed that post‐splenectomy patients tended to suffer post‐operative bleeding (0.01%, 3/246). Of the four studies^16^, ^17^, ^18^, ^19^ where the LMWH treatment was terminated immediately after bleeding, the degree of post‐operative bleeding was successfully controlled. In Haili *et al*.'s study,^14^ one patient experienced severe bleeding and required another operation. To sum up, we can reject the hypothesis that cirrhosis patients with PH and hypersplenism have a higher risk of bleeding. Under this circumstance, the use of anticoagulation or thrombolysis after splenectomy often represents a clinical challenge. Because the four studies in our meta‐analysis were retrospective non‐randomized studies, the finding of our analysis should be interpreted with caution due to the interference from factors such as the preoperative status of patients and surgeon's experience.

Our study had several limitations. First, the primary outcome proved that LMWH was effective, but the RT group and the RCT group in the process of subgroup analysis have come to a different conclusion. Generally, without randomization, it is hard to control for confounding variables, as there may have been surgeon and institution factors influencing outcomes. Second, the doses of LMWH administered to the patients were different in included studies, between 3000^19^ and 10 000 U^15^ per day. Finally, five of the six studies performed in Asian countries were included in our meta‐analysis, which might result in regional bias. Nevertheless, further multicentre studies with large patient samples are required to overcome the above‐mentioned limitations and confirm our findings.

## Conclusion

Despite these limitations, this study has clinical value. Our meta‐analysis suggested that LMWH could effectively decrease the incidence of PVST in post‐splenectomy patients without an increased risk of bleeding. However, more RCTs are warranted to further confirm the effectiveness and safety of LMWH in patients after splenectomy.

## Conflicts of interest

None declared.

## Supporting information


**Figure S1.** The Preferred Reporting Items for Systematic Reviews and Meta‐Analyses flow diagram depicting the selection process.

**Figure S2.** Meta‐analysis of the probability of portal vein system thrombosis following heparin administration after splenectomy.

**Figure S3.** Meta‐analysis of haemorrhage after splenectomy with heparin.

**Figure S4.** Egger's test for incidence of portal vein system thrombosis.

**Figure S5.** Egger's test for incidence of post‐operative bleeding.

**Table S1.** Basic characteristics of the studies included in this meta‐analysis.
**Table S2**. Summary of clinical data.
