# Supplementary figures and images for: Low‐molecular weight heparin prevents portal vein system thrombosis after splenectomy: a systematic review and meta‐analysis
Source: ANZ J Surg. 2020 Apr 27;90(12):2420–4. doi: 10.1111/ans.15865 (PMC7818250; doi:10.1111/ans.15865)

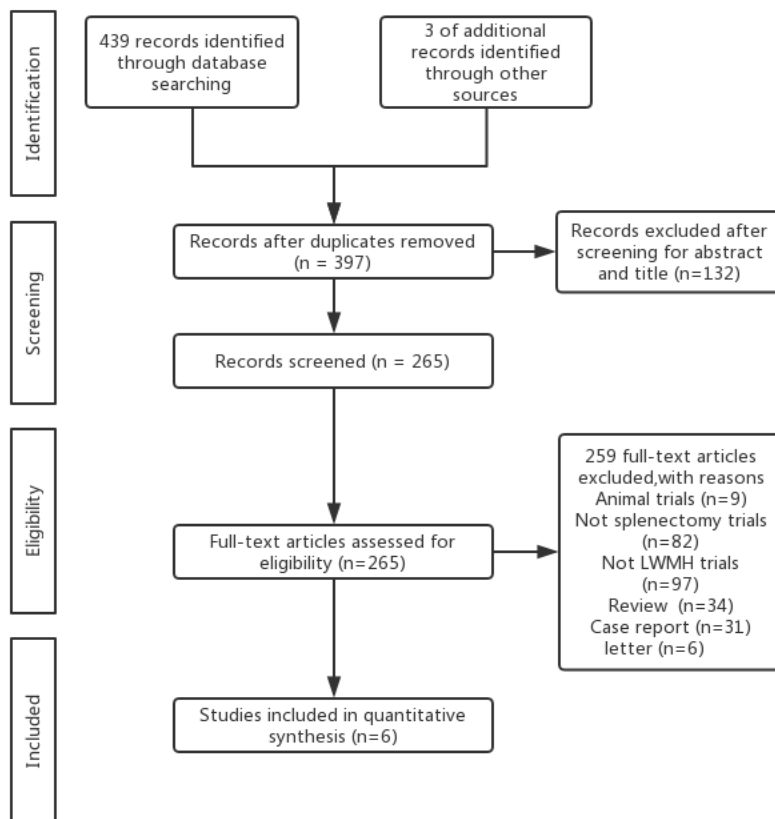

Supplement: Supplementary file 1 — Figure S1. The Preferred Reporting Items for Systematic Reviews and Meta‐Analyses flow diagram depicting the selection process. [file ANS-90-2420-s001.pdf]

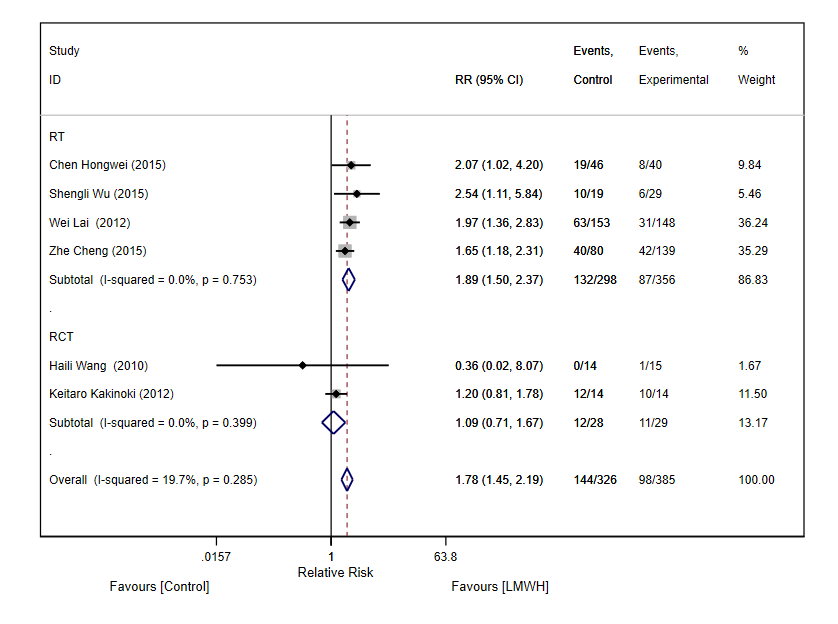

Supplement: Supplementary file 2 — Figure S2. Meta‐analysis of the probability of portal vein system thrombosis following heparin administration after splenectomy. [file ANS-90-2420-s002.tif]

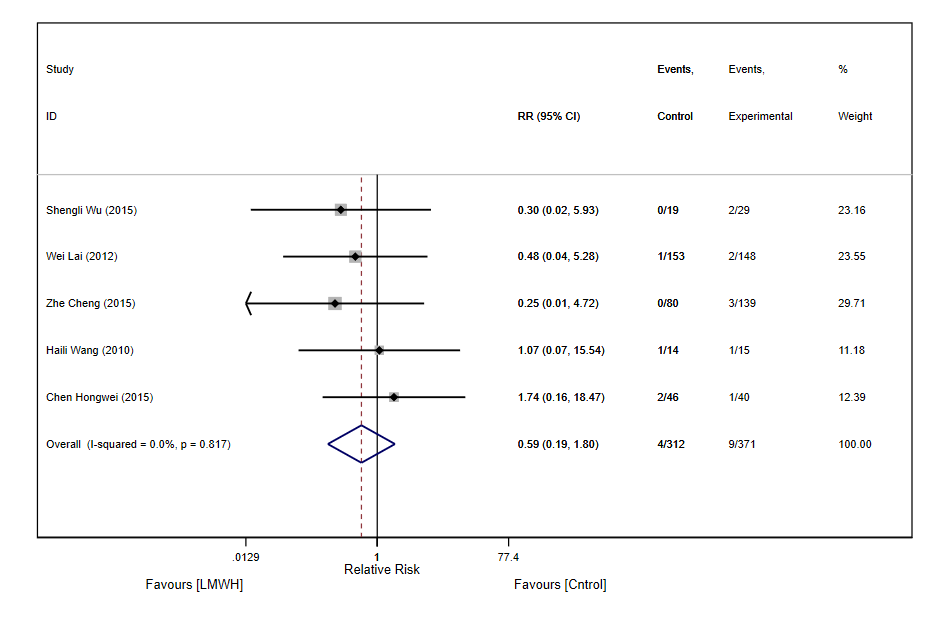

Supplement: Supplementary file 3 — Figure S3. Meta‐analysis of haemorrhage after splenectomy with heparin. [file ANS-90-2420-s003.tif]

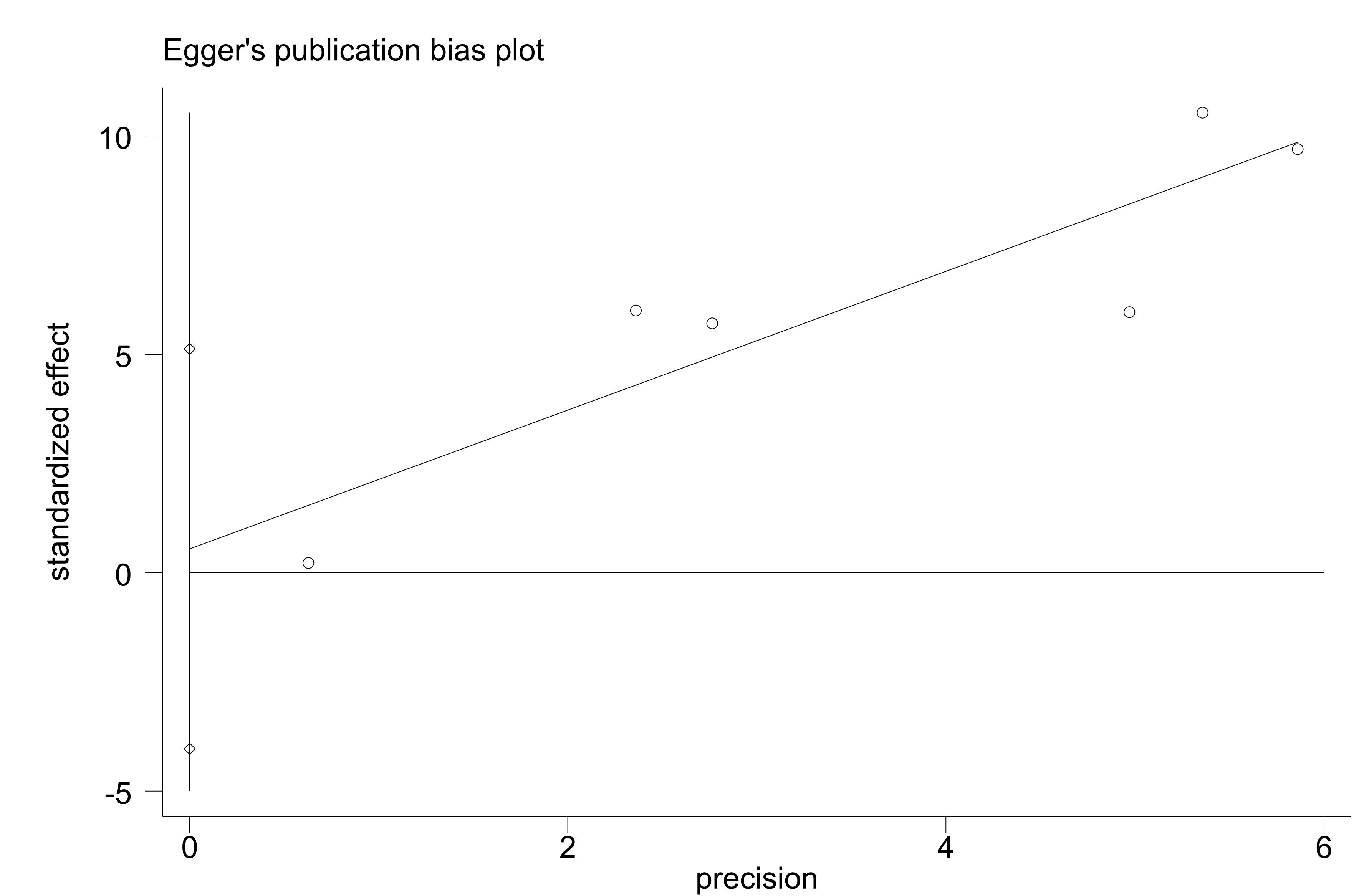

Supplement: Supplementary file 4 — Figure S4. Egger's test for incidence of portal vein system thrombosis. [file ANS-90-2420-s004.tiff]

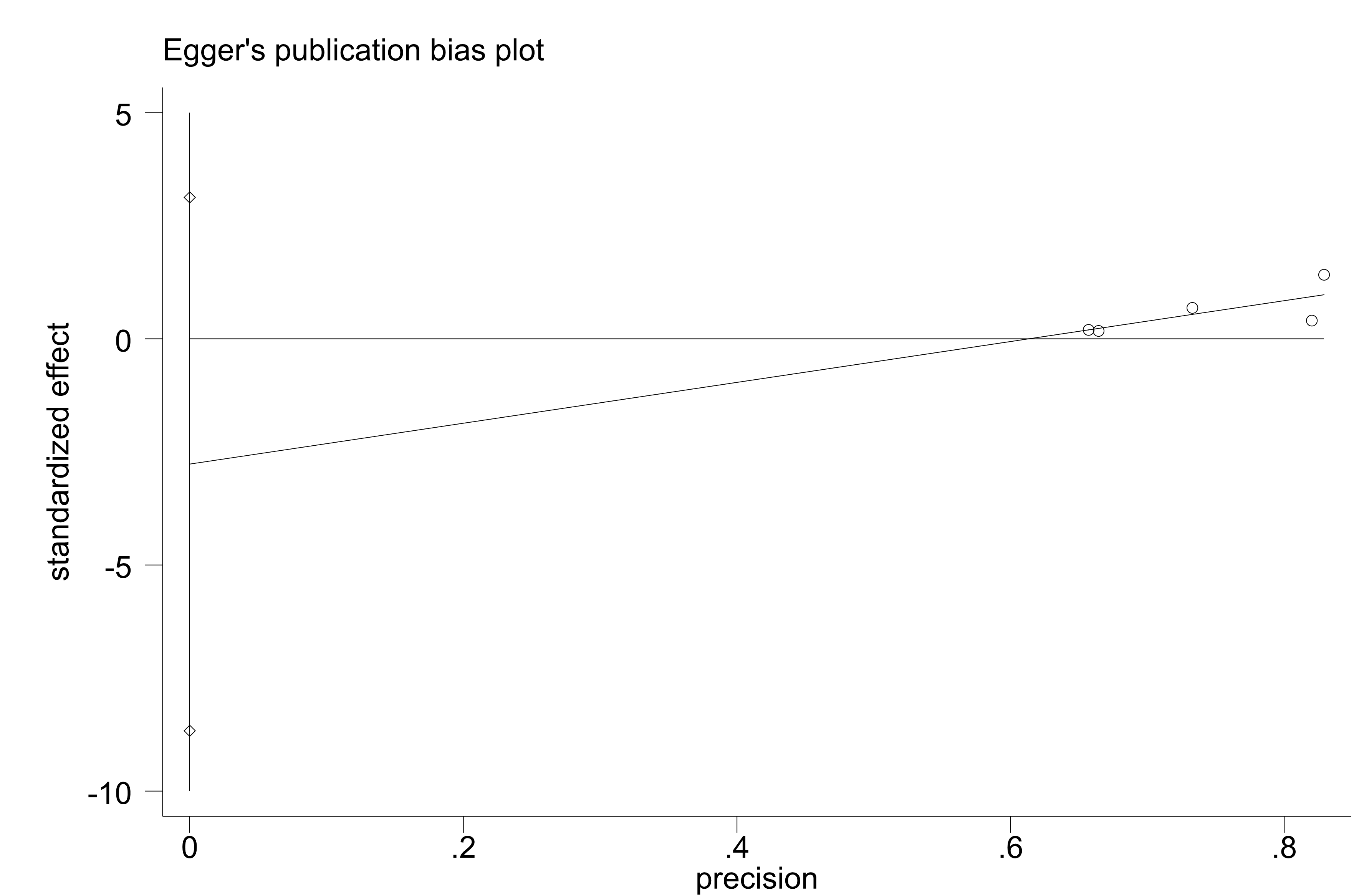

Supplement: Supplementary file 5 — Figure S5. Egger's test for incidence of post‐operative bleeding. [file ANS-90-2420-s005.tiff]
