# Supplementary material for: Low‐molecular weight heparin prevents portal vein system thrombosis after splenectomy: a systematic review and meta‐analysis
Source: ANZ J Surg. 2020 Apr 27;90(12):2420–4. doi: 10.1111/ans.15865 (PMC7818250; doi:10.1111/ans.15865)
Supplement: Supplementary file 6 — Table S1. Basic characteristics of the studies included in this meta‐analysis. Table S2. Summary of clinical data. [file ANS-90-2420-s006.docx]

**Table S1. Basic characteristics of the studies included in this meta-analysis.**

| Author, Year | | Country | Design | | | Group | Age (years) | Gender (Male/Female) | | | Etiology | Child-Pugh classification (A/B) | PPlt (×109/L) | | Portal vein diameter (mm) | Splenic vein diameter(mm) |
| --- | --- | --- | --- | --- | --- | --- | --- | --- | --- | --- | --- | --- | --- | --- | --- | --- |
| Haili[14], 2010 | Canada | | | RCT | Enoxaparin (15) | | 59 (41-61) | 11:4 | | | ITP; TTP; CLL;  Lymphoma; HS | - | 172 (120-224) | | - | - |
|  |  | | |  | Control (14) | | 43 (31-55) | 5:9 | | |  | - | 196 (87-304) | | - | - |
| Wei Lai[19], 2012 | China | | | RT | LMWH (148) | | 46.14±10.39 | 99:49 | | HBV; HCV; AC; PBC | | 125:23 | - | 12.38±1.17 | | 9.85±1.69 |
|  |  | | |  | Aspirin or warfarin (153) | | 46.47±9.58 | 103:50 | |  |  | 121:32 | - | 12.90±1.20 | | 10.10±1.41 |
| Chen Hongwei[16], 2015 | China | | | RT | LMWH (40) | | 49.4±14.3 | 26:14 | | | HBV; HCV; AC; AIC | 25:15 | 29.8±11.3 | | 15.8±2.4 | 14.5±3.6 |
|  |  | | |  | Control (46) | | 47.7±11.3 | 30:16 | | |  | 32:14 | 28.8±11.2 | | 15.2±2.7 | 14.4±3.7 |
| Shengli Wu[18], 2015 | China | | | RT | Control (19) | | - | - | | HBV; HCV; | | - | - | | - | - |
|  |  | | |  | LMWH (29) | | - | - | |  |  | - | - | | - | - |
| Keitaro Kakinoki[15], 2012 | Japan | | | RCT | LMWH (14) | | - | - | | HBV; HCV; AC; AIC | | - | - | | - |  |
|  |  | | |  | Control (14) | | - | - | |  |  | - | - | | - |  |
| Cheng[17], 2015 | China | | | RT | LMWH (139) | | 48.5±12.2 | 106:33 | HBV; HCV; AC; AIC  ILC | | | 45:94 | 16.8±6.6 | | 13.6±1.9 | 21.1±5.8 |
|  |  | | |  | Control (80) | | 45.6±13.8 | 63:17 |  |  |  | 34:46 | 17.1±6.7 | | 13.9±1.5 | 23.1±6.7 |

RT: retrospective nonrandomized trial; RCT: randomized controlled trial; LMWH: low molecular weight heparin; Control: without LMWH; "-": the data were not available; HBV: hepatitis B virus-induced cirrhosis; HCV: hepatitis C virus-induced cirrhosis; AC: alcoholic cirrhosis; AIC: autoimmune liver cirrhosis; ILC: idiopathic liver cirrhosis; ITP: immune thrombocytopenic purpura; TTP: thrombotic thrombocytopenic purpura; CLL: chronic iymphocytic leukemia; HS: hereditary spherocytosis; PBC: primary biliary cirrhosis; PPLT: preoperative platelet count

| **Author, Year** | **Operation** |  | **PVST incidence (n, %)** | **Bleeding incidence (n)** | **PVST location** | **Bleeding location** | **Detection medium** |
| --- | --- | --- | --- | --- | --- | --- | --- |
| **Haili[14], 2010** | **LS** |  | **1 (0.67%)** | **2** | **SV (1)** | **-** | **DU** |
|  |  |  | **0** | **0** | **-** | **-** |  |
| **Wei Lai[19], 2012** | **Splenectomy with GD** |  | **31 (20.9%)** | **2** | **PV (18); SV (4); Both PV and SV (7)** | **GI bleeding** | **DU** |
|  |  |  | **63 (41.1%)** | **1** | **PV (31); SV (12); Both PV and SV (19); SMV (1)** | **GI bleeding** |  |
| **Chen Hongwei[16], 2015** | **Splenectomy with GD** |  | **8 (20.0%)** | **1** | **PV (4); SV (2) ;SMV (1); IMV (1)** | **-** | **DU** |
|  |  |  | **19 (41.3%)** | **2** | **PV (12); SV (4); SMV (2); IMV (1)** | **-** |  |
| **Shengli Wu[18], 2015** | **Splenectomy** |  | **6 (20.7%)** | **2** | **PV (1); SV (5)** | **Epistaxis** | **DU** |
|  |  |  | **10 (52.6%)** | **0** | **PV (2); SV (1); SMV (2);PV+SV(3);PV+SV+SMV(1)** | **Subcutaneous ecchymosis** |  |
| **Keitaro Kakinoki[15], 2012** | **HLS** |  | **10 (71.4%)** | **-** | **-** | **-** | **DU** |
|  |  |  | **12 (85.7%)** | **-** | **-** | **-** |  |
| **Cheng[17], 2015** | **LS with GD** |  | **42 (30.2%)** | **3** | **-** | **Abdominal bleeding** | **DU with upper abdominal CTA** |
|  |  |  | **40 (50.0%)** | **0** | **-** | **Abdominal bleeding** |  |

**Table S2.** **Summary of clinical data**

LP: laparoscopic splenectomy; GD: gastroesophageal devascularization; HLS: hand-assisted laparoscopic splenectomy; DU: Doppler ultrasonography; CTA: CT angiography; PV: portal vein; SV: splenic vein; SMV: superior mesenteric vein; IMV: inferior mesenteric vein; GI: gastrointestinal
